# The Oncogenic Roles of JC Virus T Antigen in Breast Carcinogenesis

**DOI:** 10.3389/fmolb.2021.687444

**Published:** 2021-08-12

**Authors:** Hua-chuan Zheng, Ying E, Zheng-guo Cui, Shuang Zhao, Yong Zhang

**Affiliations:** ^1^ Department of Oncology and Experimental Center, The Affiliated Hospital of Chengde Medical University, Chengde, China; ^2^ Department of Oncology, Liaoning Cancer Hospital, Shenyang, China; ^3^ Department of Environmental Health, University of Fukui School of Medical Science, Fukui, Japan; ^4^ Department of Pathology, Liaoning Cancer Hospital, Shenyang, China

**Keywords:** JC virus T antigen, oncogenesis, breast cancer, dysplasia, pathological behaviors

## Abstract

**Purpose:** JC virus (JCV) infects 80–90% of the population and results in progressive multifocal leukoencephalopathy upon immunodeficiency. The study aimed to pathologically clarify the oncogenic roles of T antigen in human breast cancers.

**Methods:** Breast cancer, dysplasia, and normal tissues were examined for *T antigen* of JCV by nested and real-time PCR. The positive rate or copy number of T antigen was compared with clinicopathological parameters of breast cancer. JCV existence was morphologically detected by immunohistochemistry and *in situ* PCR. T antigen was examined by Western blot using frozen samples of breast cancer and paired normal tissues.

**Results:** According to nested PCR, the positive rate of breast ductal or lobular carcinoma was lower than that of normal tissue (*p* < 0.05). T antigen existence was negatively correlated with E-cadherin expression and triple-negative breast cancer (*p* < 0.05), but positively correlated with lymph node metastasis and estrogen receptor and progestogen receptor expression (*p* < 0.05). Quantitative PCR showed that JCV copies were gradually decreased from normal, dysplasia to cancer tissues (*p* < 0.05). JCV T antigen copy number was lower in ductal adenocarcinoma than in normal tissue (*p* < 0.05), in line with in situ PCR and immunohistochemistry. JCV copies were negatively correlated with tumor size and E-cadherin expression (*p* < 0.05), but positively correlated with G grading of breast cancer (*p* < 0.05). Western blot also indicated weaker T antigen expression in breast cancer than normal tissues (*p* < 0.05).

**Conclusion:** JCV T antigen might play an important role in breast carcinogenesis. It can be employed as a molecular marker for the differentiation and aggressive behaviors of breast cancer.

## Introduction

Breast cancer is the leading cause of cancer death among women, even in less developed countries (Coughlin, 2019). Environmental, hereditary, and genetic backgrounds are considered as the most important factors for carcinogenesis and the subsequent progression of breast cancer. Among environmental factors, viral infection is associated with about 15–20% of all cancers (FahadUllah, 2019).

JC virus (JCV) is a member of the polyomavirus family (Assetta and Atwood, 2017) and has a circular and double-stranded DNA genome. The early region encodes T antigen (Reiss and Khalili, 2003), a large phosphoprotein that binds to the viral replication region to promote double helix unwinding and recruitment of DNA synthesis proteins. The late region encodes the capsid structural proteins VP1, VP2 and, VP3 and agnoprotein (Reiss and Khalili, 2003; Saxena et al., 2021). Serologically, there is asymptomatic JCV infection in 80–90% of the adult population (Ahye et al., 2020). JCV enters the human body through both digestive and respiratory tracts and persists quiescent in the kidney and lymphoid tissues (White and Khalili, 2005; Delbue et al., 2017; Dwyer et al., 2021). However, it may be activated under immunosuppressive conditions, resulting in progressive multifocal leukoencephalopathy (PML) (Reiss and Khalili, 2003; Delbue et al., 2017; Ahye et al., 2020; Dwyer et al., 2021; Saxena et al., 2021). During permissive infection, replication of viral DNA can cause lytic infection, but in non-permissive cells, either abortive infection or cell transformation is the outcome (Delbue et al., 2017; Ahye et al., 2020; Saxena et al., 2021). JCV can transform cells, which display rapid growth and division, prolongation of lifespan, anchorage-dependent growth, chromosomal instability, and dense foci formation in culture (Eash et al., 2004; White and Khalili, 2005).

Intravenous or intracranial inoculation of JCV has been found to cause astrocytomas, glioblastomas, neuroblastomas, and medulloblastomas (Miller et al., 1984; Hayashi et al., 2001). In addition, transgenic mice expressing T antigen developed pituitary adenomas or malignant peripheral nerve sheath tumors (London et al., 1978; Del Valle and Khalili, 2021). In previous studies, we established transgenic mice and found that T antigen induced lens tumors and lung cancer (Gou et al., 2015; Noguchi et al., 2013). Recently, JCV was demonstrated to correlate with colorectal, gastric, prostatic, and esophageal cancers, brain tumors, lung cancer, and B cell lymphomas (Del Valle et al., 2005; Zheng et al., 2007a; Murai et al., 2007; Kutsuna et al., 2008; Ramamoorthy et al., 2011; Shen et al., 2011; Wang et al., 2012; Noguchi et al., 2013; Anzivino et al., 2015; MalekpourAfshar et al., 2016). The oncogenesis of JCV mainly centers on T antigen. T antigen can inactivate p53 and Rb and disrupt the Wnt signaling pathway (Piña-Oviedo et al., 2006). The encoding genes of JCV are known to be preferentially expressed in astrocytes and oligodendrocytes of the human brain because these cells contain specific transcriptional factors (NF-1, Sp1, Sμbp-2, and Purα) (Piña-Oviedo et al., 2006). Reportedly, investigators have identified JCV in the liver, kidney, spleen, bone marrow, bladder, prostate, and tonsils by PCR (Zheng et al., 2009). Here, we examined the existence and copy number of JCV in breast cancer, dysplasia, and normal tissues by nested PCR, real-time PCR, immunohistochemistry, and *in situ* PCR and compared JCV existence with clinicopathological parameters of breast cancer.

## Materials and Methods

### Subjects

Formalin-fixed and paraffin-embedded breast cancer (n = 112), breast dysplasia (*n* = 18), and normal breast (*n* = 48) tissues were sampled from surgical materials in The Affiliated Hospital of Chengde Medical University and Liaoning Cancer Hospital. Fresh samples of breast cancer and paired normal tissues (*n* = 10) were collected from both hospitals. The cancer patients did not receive a neoadjuvant before surgical operation. They signed informed consent. The ethics committees of these hospitals approved the study.

### DNA Extraction and Checking

Paraffin-embedded blocks were incised into 10-μm-thick sections, microdissected under the guidance of HE slides, and subjected to deparaffinization and rehydration. DNA was extracted by the traditional proteinase K/phenol/chloroform method. To check DNA integrity, we amplified tissue DNA by targeting β-globin: Forward; 5′-ACA​CAA​CTG​TGT​TCA​CTA​GC-3′; backward; 5′-GTC​TCC​TTA​AAC​CTG​TCT​TG-3′. PCR condition was described as follows: 30 cycles of denaturation at 95°C for 20s, annealing at 55°C for 35 s, and extension at 72°C for 20 s. DNA-free amplification was employed as a negative control.

### Nested PCR

PCR was carried out by targeting *T antigen.* T1 (5′-TGGCCTG TAAAGTTCTAGG CA -3′ and T2 (5′-GCA​GAG​TCA​AGG​GAT​TTA​CCT​TC-3′) primers were used for the 1st PCR, whereas T1 and T3 (5′-AGC​AAC​CTT​GAT​TGC​TTA​AGA​GA-3′) were used for the 2nd PCR. The 20 µl of reaction mixtures contained 0.1 µl of Ex Taq HS (TaKaRa) with 2.0 mM of MgCl_2_ *,* 2*.*0 µl × 10 PCR buffer, 2.0 µl of dNTP mixture, 1 µM of each primer, and 200 ng of DNA. The PCR process was 32 cycles of denaturation at 95°C for 20 s, annealing at 56°C for 20 s, and extension at 72°C for 20 s. Nested PCR was carried out using 1% (volume) of the first amplicon. DNA-free amplification was employed as a negative control.

### Real-Time PCR

SYBR fluorescence PCR was used to quantify JCV copies using the Bio-Rad PCR system. A plasmid of PBS-JCV T antigen was serially diluted for standard reference. These standard and sample DNAs were amplified by targeting *T antigen*: Forward: 5′-GCC​ACC​CCA​GCC​ATA​TAT​TG-3′ and backward: 5′-GTT​GAC​AGT​ATC​CAT​ATG​ACC​AGA​GAA-3′. In total, the 20 µl reaction mixture contained 10.0 µl of TaqMan^®^ ( × 2) with 1.8 µl (10 µM) of each primer and 80 ng of DNA. The PCR protocol was 55 cycles of denaturation at 95°C for 25 s, annealing at 55°C for 50 s, and extension at 72°C or 25 s.

### Immunohistochemistry

Serial sections were deparaffinized with xylene, rehydrated with alcohol, and subjected to antigen retrieval by irradiation in target retrieval solution (TRS, DAKO, CA, United States) for 5 min with a microwave oven. Five percent BSA was then applied to incubation for 5 min to prevent non-specific binding. The sections were incubated with anti-SV 40 T antigen antibody (Calbiochem, United States; 1:100) for 20 min and then treated with the anti-mouse Envision-PO (1:100, DAKO, CA, United States) antibody for 20 min. Incubation was performed in a microwave oven for intermittent irradiation as described previously (Gou et al., 2015). After each treatment, the slides were washed with TBST (10 mM of Tris-HCl, 150 mM of NaCl, 0.1% Tween 20) three times for 5 min. All slides were colored with 3, 3′-diaminobenzidine and counterstained with Mayer’s hematoxylin. Omission of the primary antibody was used as a negative control.

### *In Situ* PCR

A 10-μm-thick section was prepared with proteinase K for 15 min. After TBS washing, the tissue slide was fixed with 4% paraformaldehyde and then washed with 2 × SSC. After that, 100 μl of PCR mixture (0.2 μM of primers, 0.125 nM of digoxigenin-11-dUTP, 2.5 mM of MgCl_2_, 1 × PCR buffer, 6.25 U of Taq polymerase) was put into a membrane, sealing on the tissue. PCR amplification was performed at the condition: Denaturation at 94°C for 3 min, followed by 20 cycles of 92°C for 15 s, 55°C for 20 s, and 72°C for 30 s and final extension at 72°C for 7 min. The primers were forward: 5′-AGG​TAG​GCC​TTT​GGT​CTA​A-3′ and backward: 5′-TGC​CTA​GAA​CTT​TAC​AGG-3′. After that, the tissue was washed with 2 × SSC and incubated with blocking solution (100 μg/ml of Salmon testis DNA, 100 μg/ml of yeast tRNA, and 5% BSA) for 1 h. Subsequently, the sections were reacted with anti-digoxigenin and AP (alkaline phosphatase)-conjugated antibody (Roche, 1:500) for 90 min. After being washed for 5 min and immersed in solution II (100 mM of Tris-HCl, pH 9.5, 100 mM of NaCl, and 50 mM of MgCl_2_) for 15 min, the positive signal was colored using NBP/BCIP. Finally, methyl green was used for counterstaining.

### Western Blot

We homogenized breast cancer and normal tissues in RIPA lysis buffer, and a protein assay was performed using Kaumas brilliant blue. A 50 μg protein/sample was subjected to 10% SDS-PAGE electrophoresis and electrically transferred to a PVDF membrane, which was incubated with 5% bovine serum albumin (BSA) in TBST, and then with anti-SV40 T antigen (1:200; Santa Cruz) or rabbit anti-GAPDH (1:2,000, CST) antibody. The membranes were washed with TBST and incubated with anti-rabbit or anti-mouse HRP-conjugated secondary antibody (DAKO, 1:1,000). Bands were visualized with Azure Biosystem C300 by an ECL detection kit.

### Statistical Analysis

SPSS v. 26.0 software was used for statistical analysis. Statistical analysis was carried out using Fisher’s test for the comparison of positive rates and Mann–Whitney *U* for the comparison of means. A *p* value < 0.05 was statistically regarded as significant.

## Results

**FIGURE 1:**
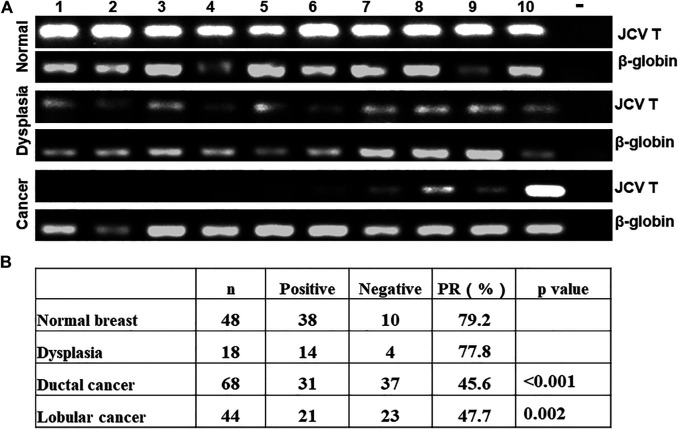
Detection of JCV T antigen in breast carcinogenesis. β-globin was positive in all cases of normal tissue, dysplasia, and cancer of the breast. T antigen was positive in some cancer cases by nested PCR **(A)**. The positive rates of JCV T antigen were compared between cancer, dysplasia, and normal tissue **(B)**. -, negative control; PR, positive rate.

As indicated in Figure 1A, we used general PCR of β-globin as quality control. We observed positive bands in all samples. Nested PCR indicated that the positive rate of T antigen was lower in breast ductal or lobular carcinoma than normal tissues (Figure 1B, *p* < 0.05). As shown in Table 1, T antigen existence was negatively correlated with E-cadherin expression (64.3 vs. 30.3%, *p* < 0.05) and triple-negative breast cancer (TNBC, 52.8 vs. 18.2%, *p* < 0.05), but positively correlated with lymph node metastasis (42.9 vs. 64.7%, *p* < 0.05), estrogen receptor (ER) expression (25.0 vs. 55.0%, *p* < 0.05), and progestogen receptor (PR) expression (26.1 vs. 55.8%, *p* < 0.05).

**TABLE 1:** The correlation between JCV T antigen existence and clinicopathological features of breast cancer.

Parameters	n	JCV T antigen		χ2	*p* value
		-	+		
N staging	97			4.217	**0.040**
-		36 (57.1%)	27 (42.9%)		
+		12 (35.3%)	22 (64.7%)		
E-cadherin expression	61			7.044	**0.008**
-		10 (35.7%)	18 (64.3%)		
+		23 (69.7%)	10 (30.3%)		
ER expression	100			5.762	**0.016**
-		15 (75.0%)	5 (25.0%)		
+		36 (45.0%)	44 (55.0%)		
PR expression	100			6.275	**0.012**
-		17 (73.9%)	6 (26.1%)		
+		34 (44.2%)	43 (55.8%)		
TNBC	101			4.697	**0.030**
-		42 (47.2%)	47 (52.8%)		
+		9 (81.8%)	2 (18.2%)		

ER, estrogen receptor; PR, progesterone receptor; TNBC, triple-negative breast cancer.

**FIGURE 2:**
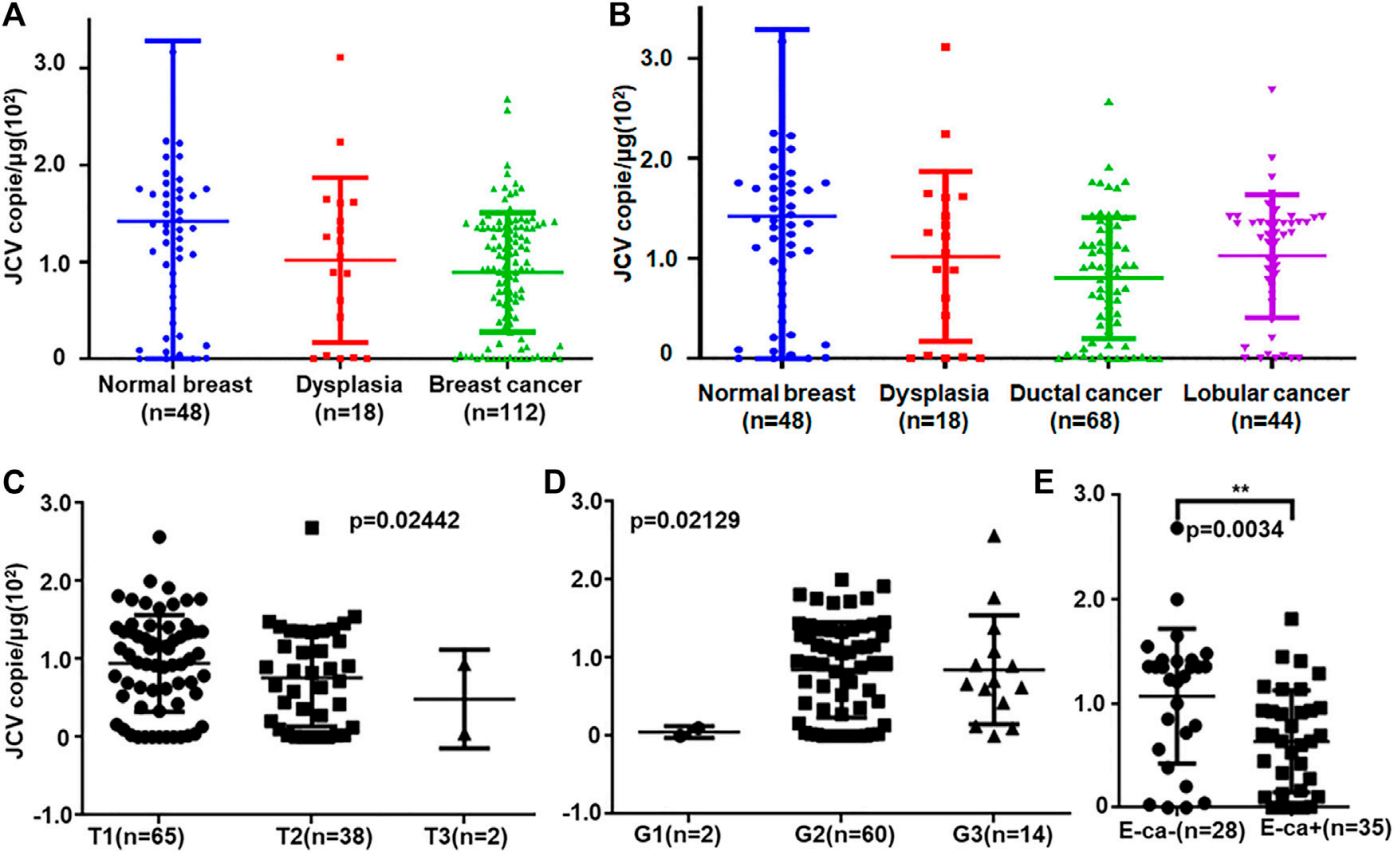
JCV T antigen loads in breast cancers. T antigen copies were measured and calculated in normal, dysplasia, and cancer tissues by real-time PCR **(A)**. The copies were analyzed between ductal and lobular cancers **(B)**. They were also compared with T staging **(C)**, G grading **(D),** and E-cadherin expression **(E)**. E-cad, E-cadherin expression.

**FIGURE 3:**
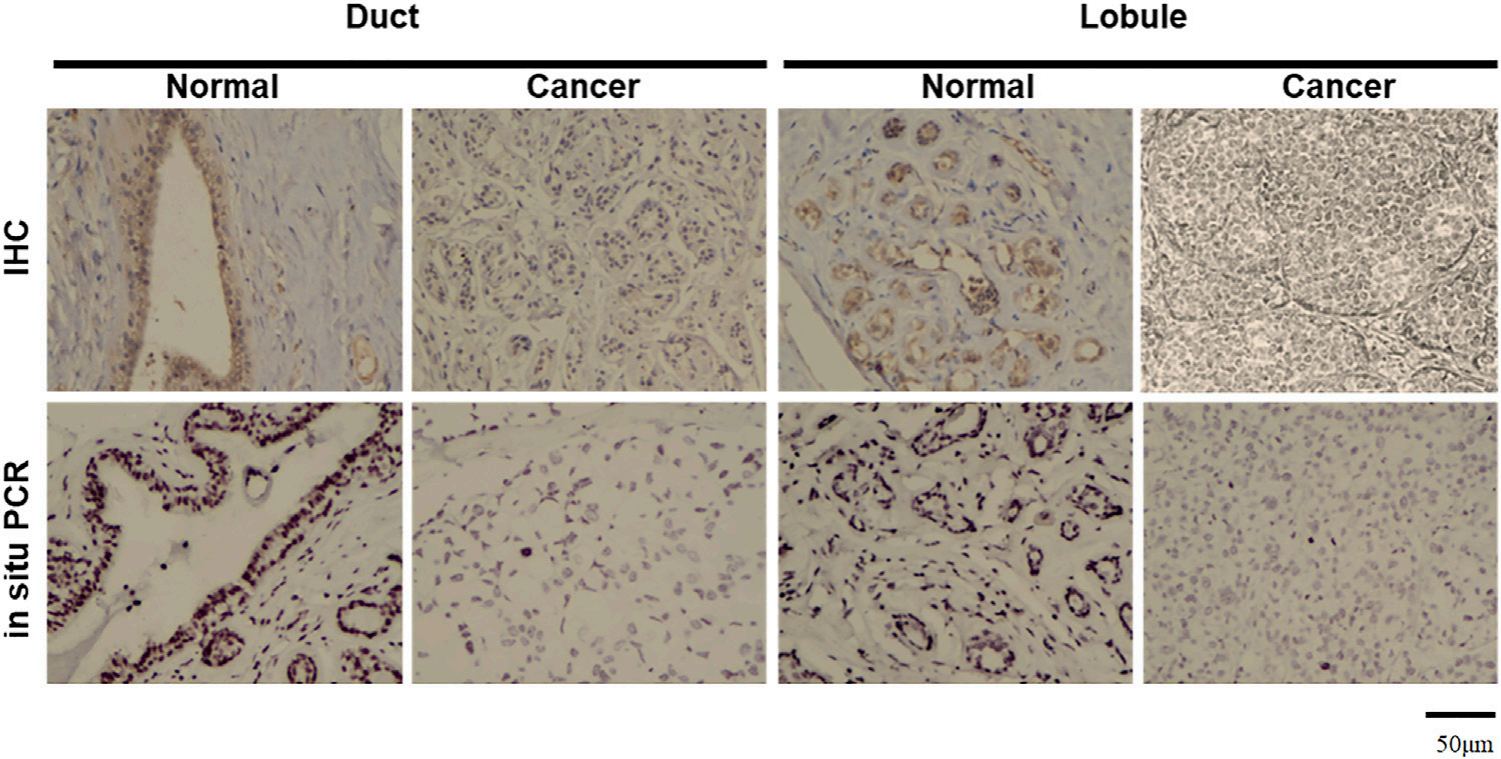
Morphological examination of JCV T antigen in breast cancers. According to immunohistochemistry (IHC), T antigen was strongly expressed in ductal and lobular epithelial cells (brown) but positively or weakly expressed in breast ductal and lobular cancer cells. NBT/BCIP coloring is displayed as black to show positive signals in breast ductal and lobular epithelial cells by *in situ* PCR, while methyl green is denoted as a green color for counterstaining.

**FIGURE 4:**
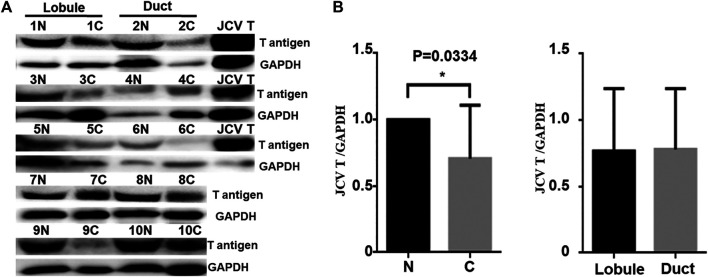
JCV T antigen expression in human breast cancers. T antigen was screened by Western blot **(A)** and subsequently analyzed by densitometric analysis **(B)**. N, normal; C, cancer. Positive control, spontaneous breast cancer from JCV T antigen transgenic mouse.

Real-time PCR showed that T antigen copies were gradually decreased from normal, dysplasia to cancer tissues (Figure 2A, *p* < 0.05). They were lower in ductal adenocarcinoma than in normal tissues (Figure 2B, *p* < 0.05). They were negatively correlated with tumor size (Figure 2C, *p* < 0.05) and E-cadherin expression (Figure 2E) and positively correlated with G grading of breast cancer (Figure 2D, *p* < 0.05). *In situ* PCR demonstrated that positive cells were detectable in breast ductal and lobular epithelium, but no or weak signal was seen in breast cancer, in line with immunohistochemistry (Figure 3). Meanwhile, a weaker T antigen expression was seen in breast cancer than that in paired normal tissues by Western blot (Figure 4).

## Discussion

Cellular malignant transformation needs a series of genetic or epigenetic accumulation, including oncogene activation or overexpression (Del Valle and Khalili, 2021). Although JCV is a highly neurotropic virus and induces brain tumors, JCV DNA was discovered in the respiratory and the upper and lower gastrointestinal tracts (Ahye et al., 2020). Furthermore, detection of JCV in renal tubules and tonsil lymphocytes indicated JCV persistence in a quiescent state of these cells during latency and infection of other cells upon immune suppression (Zheng et al., 2007b). Upon its entrance into cells, T antigen can inactivate nuclear p53 and pRb proteins to disrupt the cell cycle (Khalili et al., 2006). It can also suppress the Wnt pathway *via* β-catenin degradation and disrupt cellular IGF-IR signaling pathways (Reviriego-Mendoza and Frisque, 2011). Additionally, T antigen decreased Bag-3 expression for apoptotic suppression by blocking the interaction of AP2 with the Bag3 promoter (Sariyer et al., 2012).

Guillory et al. (2010) established a breast cancer model of the polyoma middle T antigen transgenic mouse. Tzeng et al. (1993) injected whey acidic protein (WAP)-SV-T antigen DNA into fertilized mouse eggs and found that female mice developed breast cancer with high frequency. It was very interesting that the inactivation of PTEN in CK19-positive cells caused triple-negative breast lobular carcinoma (Zhao et al., 2017). Reddi et al. (2019) reported that diffuse large B cell lymphoma appeared secondary to JCV in PML. Querido et al. (2020)observed high-grade urothelial carcinoma in a kidney transplant recipient after JCV-related nephropathy. Malhotra et al. (2016) found that JCV seropositivity was positively associated with a high lung cancer risk in the non-smoking population. Many investigators did not detect JCV in breast cancer tissue samples (Hogan et al., 1983; Antonsson et al., 2012; Dowran et al., 2019). However, Hachana et al. (2012) detected that JCV T antigen DNA was in invasive ductal carcinomas (28/112, 25.0%) but not in invasive lobular and medullary carcinomas. In this study, we found that JCV T antigen existence was gradually decreased during breast carcinogenesis at both the DNA and protein level and negatively correlated with T staging of breast cancer. No differences in T antigen DNA and protein were found in ductal adenocarcinomas or lobular adenocarcinomas. This indicated that it might be difficult for JCV infection, genomic insertion, and subsequent translation of T antigen during breast carcinogenesis and subsequent growth. However, it also cannot deny the oncogenic role of T antigen in breast cancer because it can be detected in breast cancer and its overexpression can induce breast cancer. In line with Hachana’s report (Hachana et al., 2012), we also found that JCV DNA presence correlated with TNBC. Thereby, it was believed that T antigen might be closely linked to the tumorigenesis of TNBC.

JCV T antigen was involved in colorectal carcinogenesis and liver metastasis (Sinagra et al., 2014; Shoraka et al., 2020; Vilkin and Niv, 2011). Reportedly, JCV T antigen stabilized β-catenin for its nuclear translocation to initiate cancer proliferation and development (Nosho et al., 2009). Our previous study has demonstrated that lung cancers with higher JCV copy numbers displayed high proliferation and downregulation of cell adhesion, mediated by membrane β-catenin (Zheng et al., 2007a). Donadoni et al. (2018) found that mouse T antigen-overexpressing medulloblastoma cells had survival capacity, radiation resistance, a high colony formation, and a strong double-strand DNA break repair. Noch et al. (2012) demonstrated that T antigen promoted the expression of hexokinase 2 and the pentose phosphate enzyme, transaldolase-1 for glycolysis, and pentose catabolism in medulloblastoma cells. Ksiaa et al. (2010) found that JCV presence was correlated with the patient’s age and differentiation and abnormal methylation of tumor suppressor genes of gastric cancer. Here, we found that JCV T antigen was positively correlated with G grading, N staging, E-cadherin hypoexpression, and non-TNBC, suggesting that T antigen existence might be employed to indicate poor differentiation and low E-cadherin-mediated metastasis of breast cancer.

If a virus plays an oncogenic role, it must infect the cells and then encode the oncogenic proteins to disrupt the cell function. According to our findings, we found that JCV copies were different according to tissue types (stomach < lung < breast) because the distinct distribution of its receptors (α 2, 6-linked sialic acid and serotonin) determined its different infection (Eash et al., 2004; Elphick et al., 2004; Gee et al., 2006). Geoghegan et al. (2017) showed that non-sialylated glycosaminoglycans served as alternative attachment receptors for the infection of JCV. Nukuzuma et al. (2016) found the suppressive effect of topoisomerase I inhibitors topotecan and β-lapachone on JCV propagation in human neuroblastoma cells. Adipocyte plasma membrane protein, PI3Kγ, and its regulatory subunit PIK3R5 promoted JCV infection in human glial cells (Clark et al., 2020; Haley et al., 2020). Uleri et al. (2011) showed that alternative splicing factor, SF2/ASF, negatively regulated transcription and splicing of JCV genes in glial cells. SF2/ASF hyperexpression induced growth and proliferation of JCV-transformed tumor cells. Either endogenous or ectopic LIP expression mediated the degradation of T antigen in a JCV-transgenic mouse tumor cell line (Bellizzi et al., 2015). According to our knowledge, the partner proteins of T antigen might be involved in the cell specificity of JCV T antigen, which will be confirmed in the future. Finally, estrogen and proliferation of lobular glands during breeding might increase the risk of genetic breast cancer.

In conclusion, the JCV T antigen might play an important role in breast carcinogenesis. JCV infection, insertion, transcription, translation, degradation, target, and partner chaperons of its T antigen might underlie the molecular mechanisms of its tissue-specific carcinogenesis.

## Data Availability

The original contributions presented in the study are included in the article/supplementary materials, further inquiries can be directed to the corresponding author/s.
